# Cranio-Orbito-Zygomatic Approach: Core Techniques for Tailoring Target Exposure and Surgical Freedom

**DOI:** 10.3390/brainsci12030405

**Published:** 2022-03-18

**Authors:** Sabino Luzzi, Alice Giotta Lucifero, Alfio Spina, Matías Baldoncini, Alvaro Campero, Samer K. Elbabaa, Renato Galzio

**Affiliations:** 1 Department of Clinical-Surgical, Diagnostic and Pediatric Sciences, University of Pavia, 27100 Pavia, Italy; alicelucifero@gmail.com; 2 Neurosurgery Unit, Department of Surgical Sciences, Fondazione IRCCS Policlinico San Matteo, 27100 Pavia, Italy; 3 Department of Neurosurgery and Gamma Knife Radiosurgery, IRCCS San Raffaele Scientific Institute, Vita-Salute San Raffaele University, 20132 Milan, Italy; spina.alfio@hsr.it; 4 Department of Neurological Surgery, Hospital San Fernando, Buenos Aires 1646, Argentina; drbaldoncinimatias@gmail.com; 5 Laboratory of Microsurgical Neuroanatomy, Second Chair of Gross Anatomy, School of Medicine, University of Buenos Aires, Buenos Aires 1053, Argentina; 6 Laboratorio de Innovaciones Neuroquirúrgicas de Tucuman (LINT), Facultad de Medicina, Universidad Nacional de Tucumán, Tucuman 4000, Argentina; alvarocampero@yahoo.com.ar; 7 Department of Neurosurgery, Hospital Padilla, San Miguel de Tucumán, Tucuman 4000, Argentina; 8 Department of Pediatric Neurosurgery, Leon Pediatric Neuroscience Center of Excellence, Arnold Palmer Hospital for Children, Orlando, FL 32806, USA; samer.elbabaa@gmail.com; 9 Neurosurgery Unit, Maria Cecilia Hospital, 48033 Cotignola, Italy; renato.galzio@gmail.com

**Keywords:** carotid-oculomotor window, optic-carotid window, orbitozygomatic approach, orbitopterional approach, pterional craniotomy, skull base approach, surgical anatomy

## Abstract

Background: The cranio-orbito-zygomatic (COZ) approach is a workhorse of skull base surgery, and each of its steps has a precise effect on target exposure and surgical freedom. The present study overviews the key techniques for execution and tailoring of the COZ approach, focusing on the quantitative effects resulting from removal of the orbitozygomatic (OZ) bar, orbital rim, and zygomatic arch. Methods: A PRISMA-based literature review was performed on the PubMed/Medline and Web of Science databases using the main keywords associated with the COZ approach. Articles in English without temporal restriction were included. Eligibility was limited to neurosurgical relevance. Results: A total of 78 articles were selected. The range of variants of the COZ approach involves a one-piece, two-piece, and three-piece technique, with a decreasing level of complexity and risk of complications. The two-piece technique includes an OZ and orbitopterional variant. Superolateral orbitotomy expands the subfrontal and transsylvian corridors, increasing surgical freedom to the basal forebrain, hypothalamic region, interpeduncular fossa, and basilar apex. Zygomatic osteotomy shortens the working distance of the pretemporal and subtemporal routes. Conclusion: Subtraction of the OZ bar causes a tremendous increase in angular exposure of the subfrontal, transsylvian, pretemporal, and subtemporal perspectives avoiding brain retraction, allowing for multiangled trajectories, and shortening the working distance. The COZ approach can be tailored based on the location of the lesion, thus optimizing the target exposure and surgical freedom and decreasing the risk of complications.

## 1. Introduction

The cranio-orbito-zygomatic (COZ) approach is an extension of the pterional approach, involving the adjunct of orbitozygomatic (OZ) osteotomy to allow wider exposure of the anterior and middle skull base and upper retroclival region. It provides advantages in giant aneurysms of the anterior communicating artery (ACoA) and distal basilar artery, tuberculum sellae, large anterior clinoidal, spheno-orbital meningiomas, large craniopharyngiomas, giant pituitary adenomas, cavernous hemangiomas of the hypothalamus, and crus cerebri of the midbrain. Over the years, a wide range of terms has been used to describe this approach, each of which refers to the key steps of the bony work of a specific variant. Similarly, many technical notes of the COZ approach have been reported to make its execution easier and decrease the risk of complications [1,2,3,4,5,6,7,8,9,10,11,12,13,14,15]. All these aspects have inherently been sources of confusion, thus risking excessively emphasizing the technique of execution rather than the impact that removal of the OZ complex has in increasing the angle of view of the subfrontal and subtemporal anterolateral perspectives, especially when completed with anterior and posterior clinoidectomy.

The herein presented PRISMA-based literature review strives to exhaustively summarize the spectrum of possible technical variations of the COZ approach, particularly focusing on the different quantitative effects resulting from removal of the entire OZ bar, orbital rim, and zygomatic arch. The reported data constitute the key elements for rational tailoring of the COZ approach aimed at optimizing the volume of exposure of the target, augmenting surgical freedom, and decreasing the risk of complications.

## 2. Materials and Methods 

An exhaustive literature review was performed using the PubMed/Medline (https://pubmed.ncbi.nlm.nih.gov access date 15 February 2022) and Web of Science (https://www.webofscience.com access date 17 February 2022) online databases. The keywords “cranio-orbito-zygomatic”, “orbitozygomatic”, “orbitopterional”, “zygomatic”, “orbitozygomatic”, “orbitocranial”, and “orbitofrontomalar” were combined with text words “approach”, “craniotomy”, and “osteotomy”. Articles in English or translated, without temporal restriction, were selected. Editorials, comments, case reports, and letters were excluded. Eligibility of the articles was limited to neurosurgical relevance, and the results were reported according to Preferred Reporting Items for Systematic Reviews and Meta-Analyses (PRISMA) guidelines [16]. A retrospective analysis of the personal institutional skull base pathologies register was also accomplished to select and review the cases where a COZ approach was performed, and two neurovascular illustrative cases from the personal series of the senior author (R.G.) were selected and discussed.

## 3. Results

### 3.1. Literature Volume

**Figure 1:**
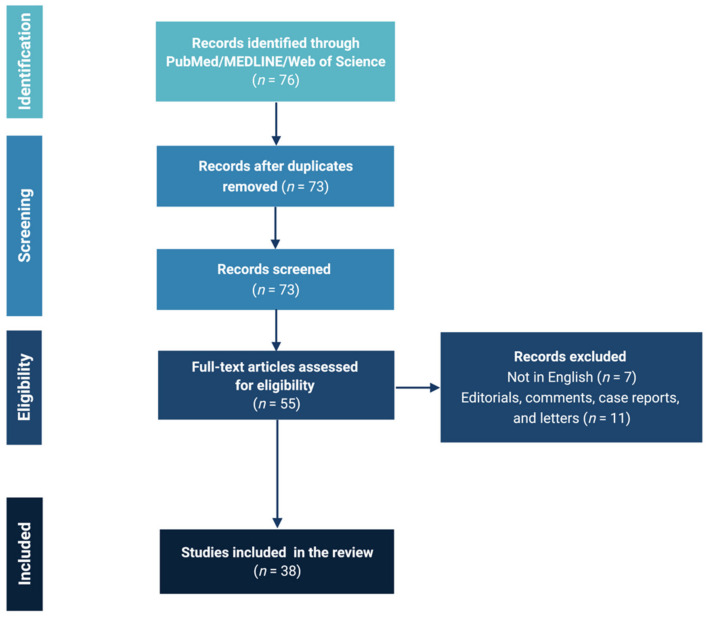
PRISMA flow chart.

Seventy-six records were initially identified. The screening led to selecting 73 eligible articles, 18 of which were furtherly excluded. A total of 55 articles were finally included in the review (Figure 1).

### 3.2. Operative Technique

#### 3.2.1. Skull Clamp Placement and Positioning

**Figure 2:**
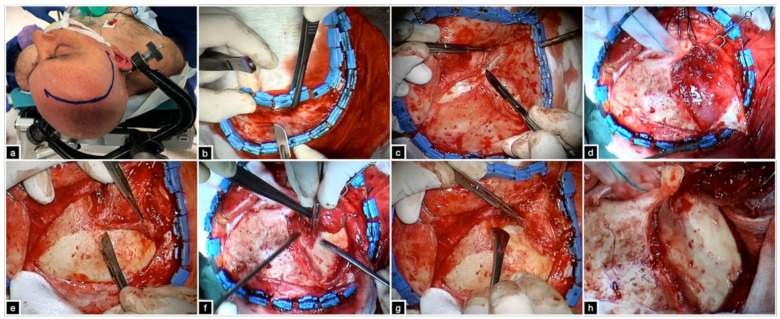
(**a**) Position and skin incision. (**b**–**d**) Galea-pericranium vascularized flap and subfascial dissection of the superficial temporal fascia. (**e**–**h**) Retrograde subperiosteal dissection of the temporalis muscle according to the Oikawa technique and skeletonization of the orbitopterional region.

The patient is placed supine with the head secured to a three- or four-point skull clamp. The trunk is elevated 20° to optimize venous outflow. The head is elevated 30° and extended 20° so that the malar eminence is the highest point of the horizon. The rotation toward the contralateral side ranges between 20° and 45° depending on the target (Figure 2a). A rotation of the head of 30° makes the longer axis of the anterior clinoid process perpendicular to the floor, while at 45°, the surgical view of the subfrontal area is maximized.

#### 3.2.2. Skin Incision and Soft-Tissue Dissection

The skin incision is made in a curvilinear fashion behind the hairline and goes from 1 cm anterior to the tragus to the contralateral midpupillary line. The skin flap is then reflected forward. The galea and periosteum are then incised together, along the superior temporal line and upper half of the skin flap, and reflected anteriorly. This galea-pericranium vascularized flap can be used for reconstruction in the case of violation of the frontal sinus. The superficial and deep layers of the superficial temporal fascia are incised jointly, 2.5 cm posteriorly to the frontozygomatic suture and 2 cm below the superior temporal line, leaving a myofascial cuff for the reconstruction (Figure 2b,c). With this subfascial technique, the anterior double-layered fascial leaflet, hosting the frontotemporal branch of the facial nerve and its interfascial fat pad, is reflected forward and the nerve remains protected (Figure 2d) [9,17,18]. The temporalis muscle is cut at the level of the frontozygomatic suture, 2 cm below the superior temporal line, to preserve the myofascial cuff. The cut curves posteriorly until the zygomatic process of the temporal bone. Subperiosteal detachment of the muscle is carried out with the Oikawa technique (Figure 2e–g) [19]. Electrocauterization must be avoided to preserve the blood supply from deep temporal arteries, preventing postoperative atrophy [19,20,21,22,23,24,25,26,27]. The periorbita is then dissected from the orbit for a depth of 3 cm to visualize the lateral aspect of the superior orbital fissure (SOF) (Figure 2h).

#### 3.2.3. Identification of Bony Landmarks

The supraorbital foramen and the supraorbital nerve are exposed. They are used as gross landmarks to identify the lateralmost limit of the air frontal sinus. Nevertheless, confirmation with neuronavigation is recommended. Identification of the coronal, sphenofrontal, sphenoparietal, sphenosquamosal, and sphenozygomatic suture is pivotal to identify the lateral third of the greater sphenoid wing. The uppermost point of the sphenosquamosal suture coincides with the superior limit of the sphenoid wing and SOF. The sphenozygomatic suture labels the midpoint of the lateral wall of the orbit. The frontozygomatic suture corresponds to the roof of the orbit.

#### 3.2.4. Craniotomy

The COZ approach can be executed in a one-piece [1,2,3,4,5,6,7], two-piece [9,10,11,12], or three-piece technique [13,14,15].

##### One-Piece Orbitozygomatic Craniotomy

**Figure 3:**
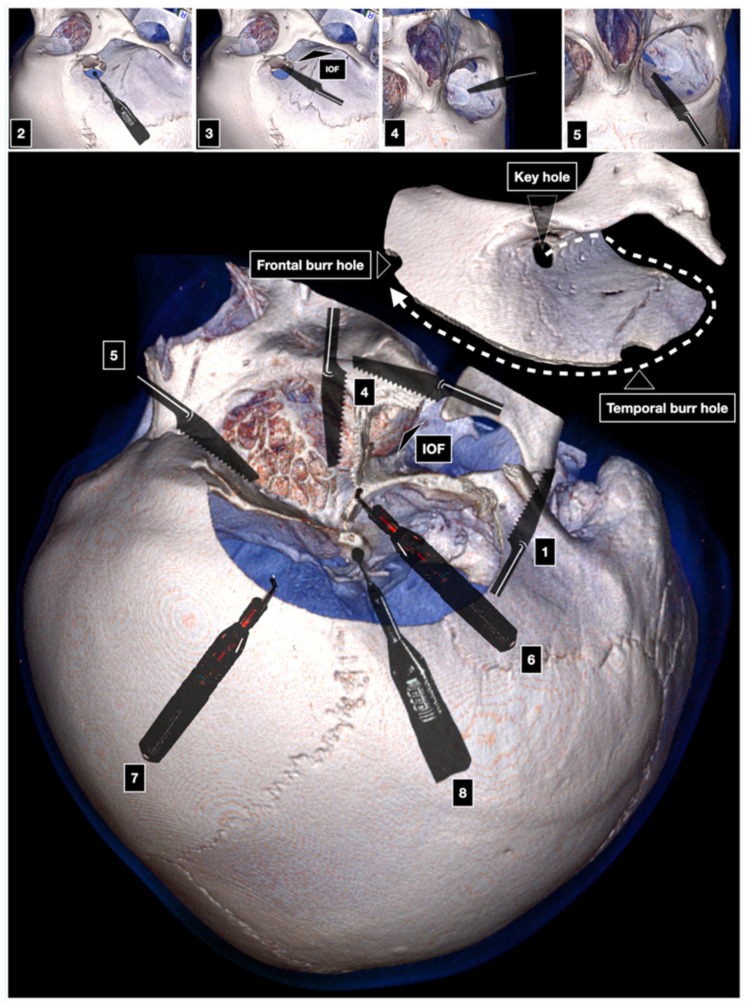
One-piece COZ approach. IOF: inferior orbital fissure. 1: first cut; 2: McCarty keyhole; 3: second cut; 4: third cut; 5: fourth cut; 6–7: connection of the keyhole with temporal and frontal burr hole; 8: anterior clinoidectomy.

The McCarty keyhole is positioned 5 mm behind the junction between the frontozygomatic, sphenozygomatic, and frontosphenoidal suture. It exposes the dura of the anterior fossa and the periorbita, along with the thin orbital roof between them [4,28]. Two further burr holes are placed in the temporal squama, above the posterior root of the zygoma, and superior temporal line, respectively. The first cut involves the posterior root of the zygoma and is carried out with a reciprocating saw. The second cut connects the keyhole to the inferior orbital fissure (IOF). The third cut starts at the level of the lateral orbital wall and is advanced across the malar eminence. The fourth cut is made at the intraorbital side and crosses the superior orbital rim and the orbital roof until the lateral aspect of the SOF. The keyhole, temporal, and frontal burr holes are interconnected with the craniotome. The COZ bone flap is then fractured by inserting and gently rotating a periosteal elevator into the groove made by the cut of the superior orbital rim (Figure 3). The temporalis muscle and the galea-pericranium flap are reflected downward. The tent stitches of the galea displace the eyeball slightly inferiorly making flatter the exposure of the anterior cranial fossa by a few millimeters. Drilling of the temporal squama significantly increases the exposure of the middle fossa, whereas drilling of the lesser sphenoid wing, along with an extra- or intradural anterior clinoidectomy, expands the surgical freedom to most targets related to this approach.

##### Two-Piece Cranio-Orbito-Zygomatic Craniotomy

The two-piece COZ approach can be executed through two different techniques, namely pterional craniotomy completed with removal of the OZ bar (Zabramski technique) and orbitopterional craniotomy also including inferior mobilization of the zygoma (Al-Mefty technique).

1. Orbitozygomatic Craniotomy (Zabramski Technique)

After the pterional craniotomy, subtraction of the OZ bar is realized through six cuts.

**Figure 4:**
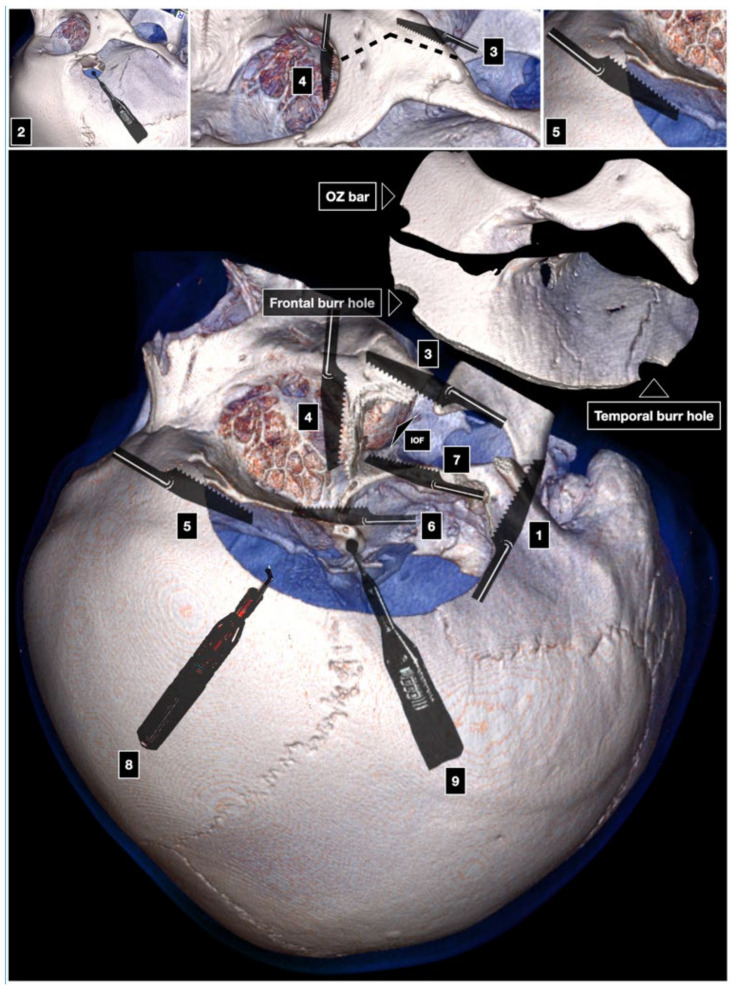
Zabramski technique of the two-piece COZ approach. OZ: orbitozygomatic. IOF: inferior orbital fissure. 1: first cut; 2: McCarty keyhole; 3: second cut; 4: third cut; 5: fourth cut; 6: fifth cut; 7: sixth cut; 8: removal of the OZ bar; 9: anterior clinoidectomy.

As in the one-piece technique, the first cut is made at the level of the posterior root of the zygoma. The second cut involves the malar eminence from lateral to medial. The third cut is carried out across the lateral orbital wall, from medial to lateral. The fourth cut is intracranial, involves the orbital roof and the superior orbital rim, and is executed starting from the lateral end of the SOF having the care to preserve the underlying periorbita. The fifth and sixth cuts connect the SOF and the IOF, thus freeing the lateral orbital wall. The fifth cut starts at the level of the IOF and ends at the level of the anterior part of the middle fossa. The sixth cut moves from the IOF toward the fifth cut (Figure 4) [11]. The OZ bar is then detached from the masseter muscle.

2. Orbitopterional Craniotomy (Al-Mefty Technique)

**Figure 5:**
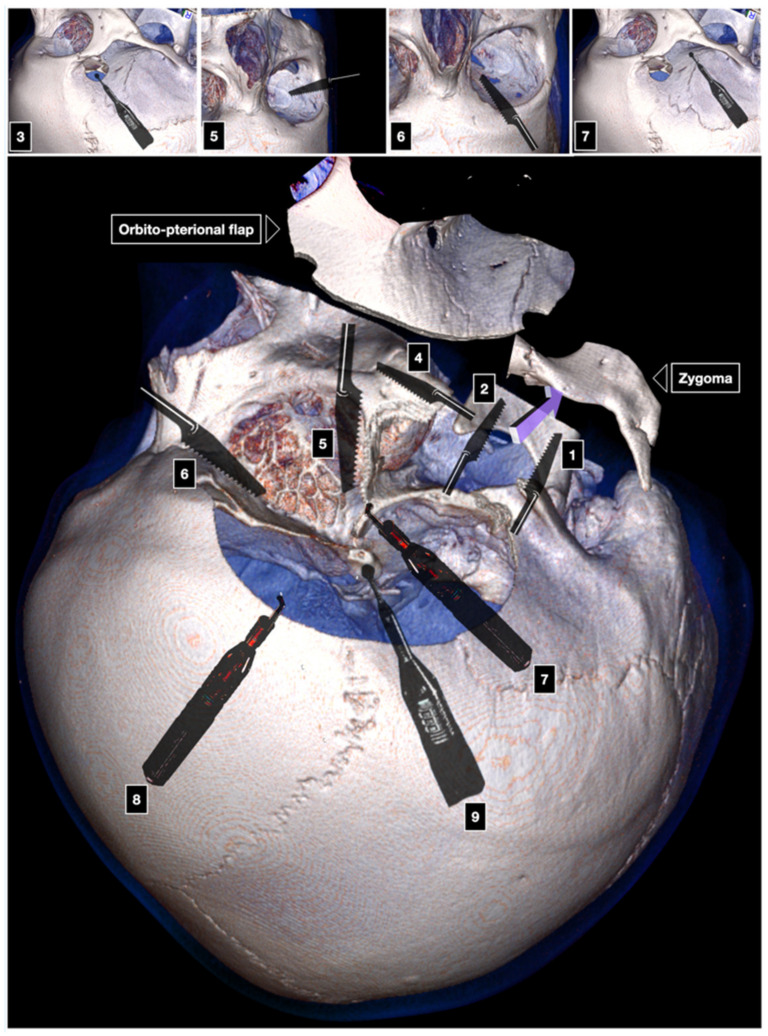
Al-Mefty technique of the two-piece COZ approach. 1: first cut; 2: second cut; 3: McCarty keyhole; 4: third cut; 5: fourth cut; 6: fifth cut; 7–8: romoval of the orbito-pterionla bone flap; 9: anterior clinoidectomy.

The orbitopterional two-piece COZ approach includes two zygomatic cuts involving the anterior and posterior root of the zygoma, respectively. The zygomatic arch is mobilized inferiorly without detachment of the masseter muscle, thus avoiding the risk of postoperative masticatory imbalance. The other steps are those of the one-piece variant of the COZ approach, where all the cuts are carried out extracranially (Figure 5) [3].

##### Three-Piece Cranio-Orbito-Zygomatic Craniotomy

**Figure 6:**
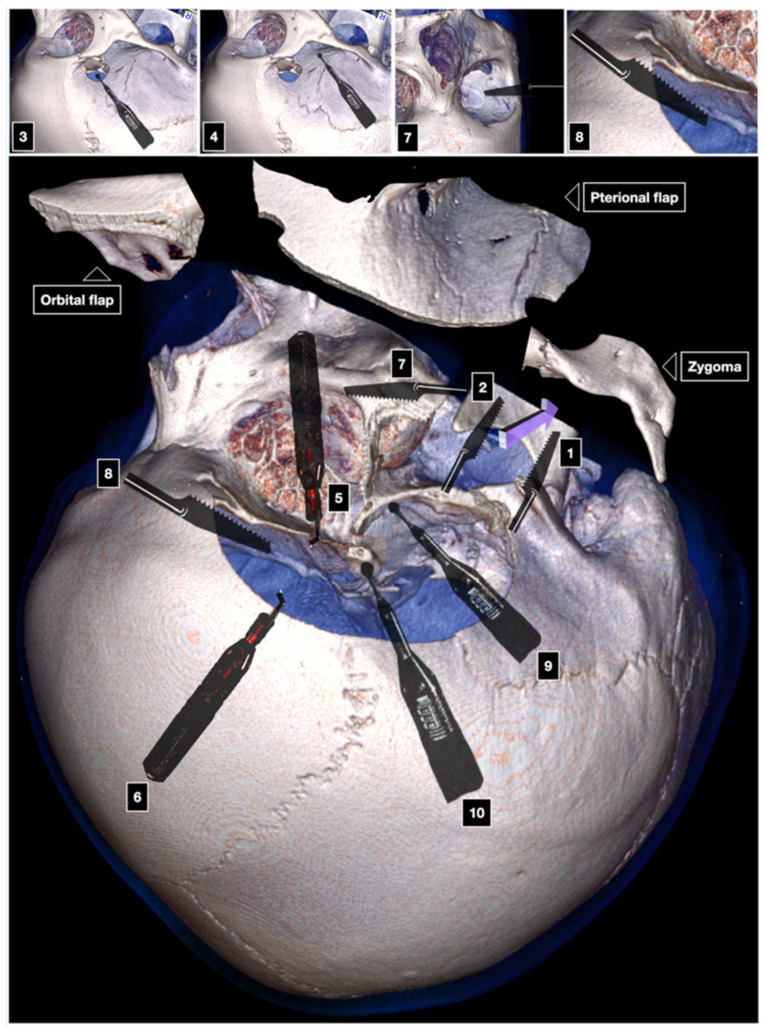
Three-piece COZ approach. 1: first cut; 2: second cut; 3: McCarty keyhole; 4: connection of the McCarty keyhole with the inferior orbital fissure; 5: elevation of the pterional bone flap; 6–8: supero-lateral orbitotomy; 9: drilling of the temporal squama; 10: anterior clinoidectomy.

The three-piece COZ approach consists of a combination of both two-piece variants. It involves zygomatic osteotomy without detachment of the masseter, inferior mobilization of the zygomatic arch, pterional bone flap, and orbital osteotomy as separate piece entailing the superolateral orbital rim and the orbital roof (Figure 6).

#### 3.2.5. Dura Opening

The dura is opened parallel to the posterior ramus of the Sylvian fissure, and two further curvilinear cuts are performed on the frontal and temporal side. This type of opening decreases the risk of damage to the Sylvian veins, especially in the case of swelling of the brain.

#### 3.2.6. Intradural Corridors

The COZ approach allows for four different corridors: (1) subfrontal, (2) transsylvian, (3) pretemporal, and (4) subtemporal. The transsylvian and pretemporal corridors are related to four well-defined deep windows to the infratentorial region through the opening of the Liliequist membrane. The deep windows are as follows: (1) optic-carotid, (2) carotid-oculomotor, (3) supracarotid, and (4) oculomotor-tentorial.

#### 3.2.7. Reconstruction

The galea-pericranium can be used as a double-layered autologous patch graft in the case of duraplasty or also if cranialization of the frontal air sinus is necessary. Preplating before proceeding to zygomatic osteotomy aids osteosynthesis and avoids the risk of postoperative facial asymmetry. Care should be taken in reconstruction of the orbital roof to limit the risk of enophthalmos. Low-profile titanium plates and 4 or 5 mm self-tapping screws are used for osteosynthesis of the bone flap. The temporalis muscle is reapproximated to the myofascial cuff at the level of the superior temporal line. An interrupted suture with 3/0 vicryl stitches is used for the superficial temporal fascia and galea. Subcutaneous drainage is left in place for 2 days to prevent blood collections. Skin suture can be optionally executed with 3/0 silk stitches or metallic agrafes.

### 3.3. Illustrative Cases

#### 3.3.1. Case 1: Complex Basilar Tip Aneurysm

**Figure 7:**
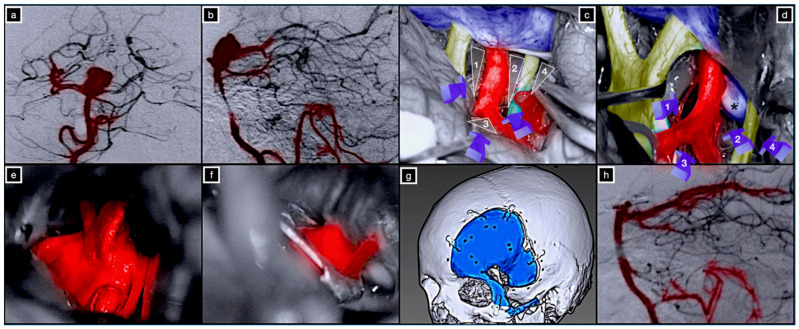
Preoperative digital subtraction angiography of the right vertebral artery in anterior–posterior (**a**) and lateral (**b**) projection. (**c**,**d**) Intraoperative pictures showing the right optic-carotid (1), carotid-oculomotor (2), supracarotid (3), and oculomotor-tentorial (4) deep windows * posterior clinoid process. Exposure (**e**) and clip ligation (**f**) of the basilar tip aneurysm. (**g**) Three-dimensional (3D) volume-rendered postoperative CT scan highlighting the COZ approach. (**h**) Postoperative digital subtraction angiography of the right vertebral artery in lateral projection.

A large basilar apex aneurysm was incidentally found in a 54-year-old female patient. On digital subtraction angiography, the aneurysm was superior projecting and presented with a dome-to-neck ratio of 1.5. Both P1 segments of the posterior cerebral arteries arose from the partially calcified neck. Basilar bifurcation was low riding, lying 8 mm below the dorsum sellae. The patient underwent a two-piece COZ approach inclusive of orbitopterional craniotomy, zygomatic osteotomy, extradural anterior clinoidectomy, lateral mobilization of the internal carotid artery (ICA), and posterior clinoidectomy. Posterior clinoidectomy expanded the carotid-oculomotor window and allowed for control of the midbasilar trunk. Given the pattern of low-riding basilar bifurcation, the additional exposure resulting from the COZ approach and posterior clinoidectomy was paramount for temporary clipping. Anterior clinoidectomy and lateral mobilization of the ICA led to expanding the optic-carotid window, which was used to dissect the neck and the thalamoperforating arteries resulting from P1, and to definitively clip the aneurysm. The transsylvian, pretemporal, and subtemporal corridors were used in combination. The patient was discharged without deficits on the fifth post-op day (Figure 7).

#### 3.3.2. Case 2: Large ACoA Aneurysm

**Figure 8:**
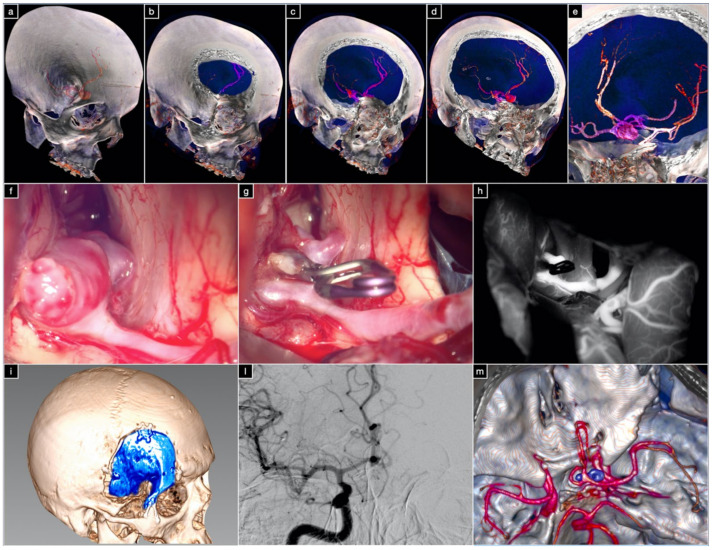
(**a**–**e**) Three-dimensional (3D) volume-rendered preoperative CT angiography. Intraoperative pictures taken before (**f**) and after (**g**) clipping of the aneurysm. (**h**) Postclipping indocyanine green videoangiography. (**i**) Three-dimensional (3D) volume-rendered postoperative CT scan showing the orbitopterional approach. (**l**) Digital subtraction angiography of the right internal carotid artery in anterior–posterior projection. (**m**) Postoperative 3D volume-rendered CT angiography.

A 48-year-old female was diagnosed with an incidental large anterior communicating artery aneurysm. The left A1 segment of the anterior cerebral artery was hypoplastic, and both A2 segments originated from the ACoA. The aneurysm was anterior–superior projecting, and the superolateral orbital rim hindered visualization of the left A2 segment. The patient underwent an orbitopterional approach, which allowed expanding the subfrontal corridor and the angular exposure of the aneurysm neck. Better visualization of the left Heubner artery and hypothalamic and subcallosal perforating arteries was also possible. The aneurysm was successfully clipped and the postoperative course was uneventful (Figure 8).

## 4. Discussion

**Figure 9:**
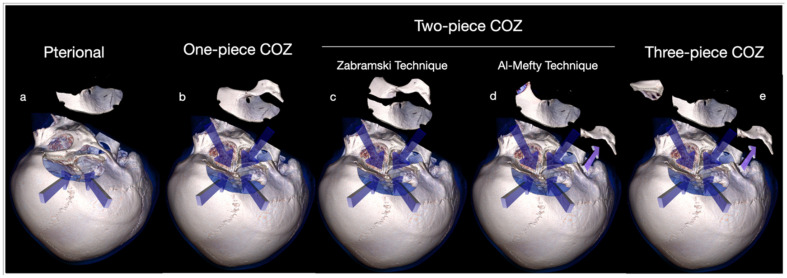
Comparison between pterional approach (**a**) and one-piece (**b**), two-piece (**c**,**d**), and three-piece (**e**) COZ approach.

The present review summarizes the key techniques for tailoring the amount of surgical exposure provided by the COZ approach, which is today considered a workhorse of skull base surgery. A gradual shift from the one-piece technique to the two-piece or three-piece technique occurred over the years (Figure 9).

The reasons consist of easier execution of the latter two variants, as well as better functional and cosmetic outcome [6,11,29,30]. Some authors also stress the safer profile of the three-piece technique deriving from the fact that the cuts of the orbital roof are performed by the intracranial side under direct vision [14]. A series of in-depth anatomical quantitative studies have clarified the effects of OZ osteotomy [31,32,33,34,35,36,37,38,39,40]. It has three main advantages, namely (1) a wider working room allowing handling the lesion from different angles of view, (2) a shorter working distance for deep neurovascular targets, and (3) an increased subfrontal and subtemporal angular exposure. These points considerably reduce the need for brain retraction, at the same time expanding tremendously the limits of the pterional route, as reported by our group [41] Among the transcranial anterolateral approaches, the COZ holds maximum surgical freedom toward a high number of deep targets because it allows concurrently combining the subfrontal, transsylvian, pretemporal, and subtemporal routes. Wide splitting of the Sylvian fissure is the key for all these corridors, which can be used in combination especially in giant or complex aneurysms as stressed by our group [15,41,42,43,44,45]. Comparing the pterional and COZ approach in terms of angular exposure and surgical freedom, Alaywan and Sindou demonstrated that removal of the OZ bar in a single block is responsible for an increase of 8°, 6°, and 10°, respectively, on the plane sagittal, coronal, and axial [36]. This additional exposure is valid for almost all lesions for which the COZ approach has indication. Angular exposure of the ACoA complex, basilar bifurcation, and posterior clinoid is increased by 75%, 46%, and 86% on average in the subfrontal, pterional, and subtemporal approaches, respectively [36]. Schwartz and colleagues reported that exposure caused by additional removal of the OZ bar is as long as the distance between the sphenoid wing and the highest point of the target on the sagittal and coronal plane (Table 1) [32].

In particular, for the basilar apex and posterior clinoid, the removal of the orbital rim and zygomatic arch as separate steps results in an increase of exposure of 28% and 22%, respectively (Table 2) [32].

**Figure 10:**
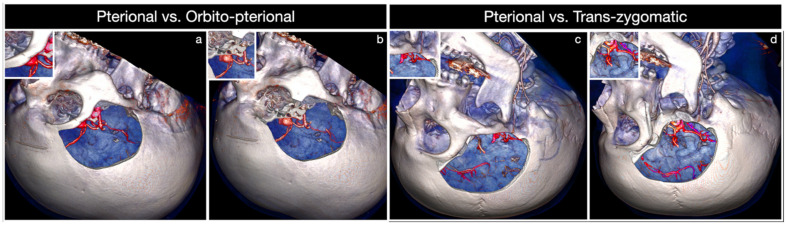
Comparison of the surgical exposure between the pterional (**a**) vs. orbitopterional (**b**) approach and the pterional (**c**) vs. trans-zygomatic (**d**) approach.

Based on these data, the COZ approach can be tailored depending on the location and size of the lesion, as well as the specific corridor deemed more suitable for the target exposure [41,46]. Many authors support OZ osteotomy exclusively for lesions projecting upward, such as high-riding ACoA and basilar tip aneurysms or intra-axial tumors abutting within the basal forebrain of the interpeduncular fossa. However, it has been proved that the increase in surgical freedom of OZ osteotomy concurs to significantly enhance the working space also for lesions extending downward [31,32,33,34,35,36,37,38,39,40]. Among the latter, a robust example is given by basilar apex aneurysms involving low-riding basilar bifurcation, as in Case 1 or voluminous parasellar tumors, especially if extended to the infratemporal fossa. The subfrontal and transsylvian perspectives are used to clip large and giant ACoA aneurysms, whereas the four deep windows related to the transsylvian and pretemporal corridors, along with the subtemporal corridor, are the main access routes to basilar tip aneurysms and ventral midbrain lesions lying in the crural and ambient cisterns [47,48,49,50,51,52]. In the COZ approach, additional exposure resulting from the OZ osteotomy is to be considered as spherical around the target rather than linear upward or downward, thus allowing for a multiangled approach to the lesion. Increasing surgical freedom related to the COZ approach in comparison with the pterional transsylvian approach is based on this key concept [41]. As specifically concerns the increase in angular exposure, superolateral orbitotomy has been reported to have quantitative and qualitative effects extremely different from those of zygomatic osteotomy [3,22,31,32,35,36,37,38,39,40,53,54,55,56,57,58,59,60]. Removal of the orbital rim, which should be considered the roof of the subfrontal perspective, significantly raises the roof of the subfrontal corridor increasing the obliquity of the line of sight backward and upward. This key step constitutes an advantage in terms of surgical freedom to the basal forebrain [8,32,37,40,53]. It also enhances the anterolateral perspective to the anterior cranial fossa through the transsylvian route (Figure 10a,b). Conversely, while not affecting the exposure of targets such as posterior clinoid, tentorial edge, and basilar apex, zygomatic osteotomy adds advantages to the pretemporal and subtemporal corridors through additional illumination of the blind spots in the depth and shortening of the working distance to the interpeduncular cistern (Figure 10c,d) [32,54].

While subtraction of the orbital rim is useful for high-riding aneurysms of the basilar apex, the shallower operative field resulting from zygomatic osteotomy enhances the pretemporal, subtemporal transtentorial, and subtemporal transpetrosal (Kawase’s) approaches in cases where the basilar tip aneurysm originates at the level of low-riding basilar bifurcation [61]. An anatomically shorter ICA narrows the optic-carotid and carotid-oculomotor window but enhances the supracarotid corridor to the interpeduncular region. Nevertheless, it should be highlighted that accessibility of the supracarotid window can be severely limited in the case of obstruction by part of the perforating arteries originating from the ICA apex. Anterior clinoidectomy, opening of the distal dural ring of the ICA, ICA mobilization, and optic foraminotomy further expand the versatility of the COZ approach for what concerns the paraclinoid region and infratentorial targets [62,63]. Lateral mobilization of the ICA can be useful to further expand the optic-carotid corridor in giant aneurysms of the basilar tip or high-riding basilar bifurcation patterns, the latter having an incidence of 30% [64,65,66,67]. On the contrary, medial mobilization of the ICA, posterior clinoidectomy, and splitting of the tentorium significantly increase the length of proximal exposure of the basilar trunk in cases where the basilar bifurcation is very low riding. This bifurcation pattern characterizes about 20% of patients [64,65,66,67].

### 4.1. Complications Avoidance

Complications of the COZ approach can be functional, aesthetic, or of both types. Some of the most frequent are injury of the frontotemporal branch of the facial nerve, atrophy of the temporalis muscle, masticatory imbalance, enophthalmos, diplopia, visual impairment, and cerebrospinal fluid leakage. The subfascial technique avoids deficit of the frontotemporal branch of the facial nerve, whereas compulsive subperiosteal blunt and “cold” (avoiding electrocauterization) dissection of the deep temporal fascia prevents postoperative atrophy of the temporalis muscle [20,24,27,41]. The subfascial technique has been reported to be safer than the interfascial technique because of anatomical variability of the frontotemporal ramus according to which, up to 30% of cases, some nerve twigs may be found within the two fascial layers [68]. Preservation of the normal trophism of the temporalis muscle is the result of some important aspects; Apart from the subperiosteal retrograde Oikawa dissection technique [19], caudal mobilization of the zygomatic arch has also been reported to decrease the risk of atrophy escaping compression of the muscle secondary to its reflection [20]. Avoidance of detachment of the masseter, along with functional preservation of the temporalis muscle, prevents the risk of masticatory imbalance. Incidence of diplopia, facial and orbital asymmetry is lessened by meticulous osteosynthesis, which is generally performed using low-profile miniplates and screws. Preplating aids in approximation of the bone flaps. Protection of the periorbita is of utmost importance to avoid enophthalmos and postoperative orbital hematoma. Risk of visual impairment or even blindness mainly comes from anterior clinoidectomy, which is added to COZ craniotomy in most cases. Risk of thermal damage to the optic nerve secondary to overheating caused by the high-speed drill must be decreased by constant irrigation. Excessive downward displacement of the eyeball during the approach is a further potential source of visual morbidity. The need to expand the subfrontal corridor frequently implies violation of the frontal sinus. Undesired communication with the sphenoid sinus can be the result of anterior clinoidectomy in cases where the anterior clinoid process is highly pneumatized. Meticulous packing of both these sites with a piece of muscle or autologous fat is recommended to dramatically reduce the risk of infections and cerebrospinal fluid leak. Reflected galea-pericranium vascularized flap constitutes a further barrier.

### 4.2. Clinical Algorithm

In the one-piece COZ approach, zygomatic osteotomy is generally less generous, involving mainly the frontal process. Therefore, the one-piece COZ approach is theoretically more suitable for parasellar and orbital tumors where wider mobilization of the zygomatic arch does not add real advantages in target exposure. 

Conversely, the one-piece COZ approach is less indicated in ruptured aneurysms, hyperostotic spheno-orbital and sphenoid meningiomas, and large intraorbital tumors with raised intraocular pressure. In ruptured aneurysms, the coexistent brain edema increases the risk of parenchymal damages during extracranial osteotomy of the orbital roof. In tumors characterized by severe hyperostosis of the roof and lateral wall of the orbit, the fracture of the one-piece bone flap can accidentally give rise to force vectors involving the optic canal and sphenoid sinus with a consequent risk of damage to the optic nerve and cerebrospinal fluid leaks. In the case of intraocular hypertension, the greater manipulation of the eyeball, equired to cut the orbital roof from the extracranial intraorbital side, increases the risk of blindness, as well as dangerous bradyarrhythmia deriving from elicitation of the trigeminal oculocardiac reflex. The two-piece variant fits most other lesions for which the COZ approach is indicated thanks to its wide intrinsic versatility that characterizes both the Zabramski and Al-Mefty techniques. The Al-Mefty technique should be preferred for malignancies affecting the temporal and infratemporal fossa since this variant avoids detachment of the masseter muscle, thus eliminating an adjunctive risk for mastication. These tumors often involve the masticatory space apart from the middle fossa. Despite its greater facility of execution, the three-piece variant is considered disadvantageous in children where the physiologic growth of the facial skeleton can be compromised by the fixation devices. Furthermore, in pediatric and extremely thin patients, where the thickness of the facial soft tissues is reduced, the greater number of miniplates and burr hole covers required for reconstruction can be sources of poor aesthetic outcome. Apart from the aforementioned aspects, which should be considered as indicative rather than imperative, the preferences and the personal experience of the surgeon also have to be considered in the choice of a specific technique of execution.

### 4.3. Limitations of the Study

The present study has some limitations that can be summarized in its retrospective nature, the intrinsic bias of the different studies involved, and the type of morphometric and quantitative studies selected which were performed only on cadavers in most cases.

## 5. Conclusions

The spectrum of variants of the COZ approach involves a one-, two-, and three-piece craniotomy, with a decreasing level of technical complexity and risk of complications.

Superolateral orbitotomy expands the subfrontal and transsylvian corridor and raises surgical freedom to the basal forebrain, hypothalamic region, interpeduncular fossa, and basilar apex.

Zygomatic osteotomy shortens the working distance to the main targets of the pretemporal and subtemporal route.

Removal of the OZ bar eliminates the need for brain retraction and allows for multiangled trajectories.

The COZ approach can be tailored based on the location and extension of the lesion, thus optimizing target exposure and decreasing the risk of complications.

## Figures and Tables

**Table 1:** Average Area of exposure of different targets according to Schwartz et al. [32].

Approach	Area of Exposure (mm^2^ ± SD)		
	Posterior Clinoid	Tentorial Edge	Basilar Tip
Pterional	2915 ± 585	2521 ± 301	1639 ± 244
Orbitopterional	3702 ± 943	3536 ± 539	2020 ± 350
COZ	4170 ± 1053	4249 ± 1186	2400 ± 386

SD: standard deviation; COZ: cranio-orbito-zygomatic approach.

**Table 2:** Percentage increase in exposure of different targets according to Schwartz et al. [32].

Approach	Surgical Target (% Increase in Exposure)		
	Posterior Clinoid	Tentorial Edge	Basilar Tip
Orbitopterional	26 *	39 *	28 *
Pterional + Zygomatic Osteotomy	13	17	22
COZ	43 *	64 *	51 *

COZ: cranio-orbito-zygomatic approach; * *p* < 0.05.

## Data Availability

All data are included in the main text.
